# Filament-Reinforced 3D Printing of Clay

**DOI:** 10.3390/ma16186253

**Published:** 2023-09-17

**Authors:** Julian Jauk, Lukas Gosch, Hana Vašatko, Markus Königsberger, Johannes Schlusche, Milena Stavric

**Affiliations:** 1 Institute of Architecture and Media, Graz University of Technology, 8010 Graz, Austria; 2 Institute of Mechanics of Materials and Structures, TU Wien, 1040 Vienna, Austria; 3 Institute of Structure and Design, University of Innsbruck, 6020 Innsbruck, Austria

**Keywords:** clay, reinforcement, 3D printing, composite material

## Abstract

This research resulted in the development of a method that can be used for filament-reinforced 3D printing of clay. Currently, clay-based elements are mixed with randomly dispersed fibrous materials in order to increase their tensile strength. The advantages of taking this new approach to create filament-reinforced prints are the increased bridging ability while printing, the increased tensile strength of the dried elements, and the achievement of non-catastrophic failure behavior. The research methodology used involves the following steps: (1) evaluating properties of various filament materials with respect to multiple criteria, (2) designing a filament guiding nozzle for co-extrusion, and (3) conducting a comprehensive testing phase for the composite material. This phase involves comparisons of bridging ability, tensile strength evaluations for un-reinforced clay prints and filament-reinforced prints, as well as the successful production of an architectural brick prototype. (4) Finally, the gathered results are subjected to thorough analysis. Compared to conventional 3D printing of clay, the developed method enables a substantial increase in bridging distance during printing by a factor of 460%. This capability facilitates the design of objects characterized by reduced solidity and the attainment of a more open, lightweight, and net-like structure. Further, results show that the average tensile strength of the reinforced sample in a dry state exhibited an enhancement of approximately 15%. The combination of clay’s ability to resist compression and the filament’s capacity to withstand tension has led to the development of a structural concept in this composite material akin to that of reinforced concrete. This suggests its potential application within the construction industry. Producing the prototype presented in this research would not have been possible with existing 3D printing methods of clay.

## 1. Introduction

Surpassing traditional methods of clay processing [1], 3D printing of ceramics started to gain ground in building technology research a few years ago [2,3], yet digital fabrication, allowing for material efficiency [4] as well as unique aesthetics [5], primarily focuses on non-sustainable materials such as concrete [6]. This study aims to address the inquiry of which structural design [7,8], material compositions, and processing techniques [9] must be devised and what basic material properties [10] must be acquired to enhance the utilization of 3D printed clay in architectural designs. Building upon research on fiber reinforcement of clay [11], interest has recently emerged in the possible industrial applications of fibrous reinforcement in the extrusion of 3D printing viscous materials, which is further discussed in this section.

So far, the addition of fibers prior to printing has been used to efficiently reinforce a material mixture on a small scale (diameter of 0.08 to 1.5 mm and a length of 2 to 20 mm) [12]. This research, however, focuses on using large-scale reinforcements (endless filament with a diameter from 0.5 to 2 mm). The following references are organized according to the used material: concrete, for which numerous examples can be found, and clay, for which fewer scientific publications exist. Furthermore, the references will be categorized by using temporal processes: pre-printing reinforcement, while-printing reinforcement, and post-printing reinforcement [13].

An example of concrete and pre-printing reinforcement is Mesh Mould, a project using a digital fabrication technique developed at the Swiss Federal Institute of Technology (ETH) in Zürich that involves an in situ welded steel reinforcement and later applied concrete [14]. This allows for the reinforcing mesh to be produced using a specific and fully automated method. The even distribution of concrete was applied manually, however, as the accessibility of the cavities within the highly complex reinforcement is low.

Pre-printing linear reinforcement of clay can be found in a project at IAAC Barcelona using tense ropes and other kinds of fiber reinforcement to strengthen 3D-printed clay. Ropes were arranged in two-dimensional layers and covered with clay. The main advantage of using this method was that it enabled the user to apply pre-tension to the reinforcement. However, this approach requires the use of formwork to tighten the ropes and manual effort [15]. Another student’s project at IAAC presents a method that can be used for 3D printing around or on top of Polylactide (PLA) structures so that they can be used as initial support structures. It was envisioned that two robots work in parallel: one 3D printing a PLA reinforcing structure and the other one printing clay around it [16]. In this way, the geometry of the reinforcement can be designed to have a specific complex and optimized shape.

The use of concrete and post-printing reinforcement is commonly utilized in commercially available methods to reinforce 3D-printed concrete. This typically involves the initial printing of outer wall segments, the placement of steel grids, and the subsequent casting of concrete to fill all recesses [17]. This method is already practically applied, as this process does not include any other digital fabrication processes than 3D printing concrete. This method of fabrication, however, also involves manual intervention.

An exemplary use of clay and post-printing was not found in ongoing research, but such examples can be found when examining the existing production techniques, e.g., filling hollow chambers of conventional masonry bonds with further structural elements or tie rods.

Incorporating concrete and while-printing techniques, an active reinforcement entrainment device called RED was developed at the Eindhoven University of Technology to enhance steel cable reinforcement in printed concrete [18]. This makes using a single automated fabrication process possible. However, the authors also reported substantial defects in the concrete matrix, particularly underneath the reinforcement cable, and efforts that need to be made to improve the bond between the two material components.

Depending on the material and the nature of the extrusion tool, a specialized technical solution is required to achieve reinforcement during the printing process [19,20]. Concurrently with the creation of this paper, a similar project was initiated at RWTH Aachen University, which successfully implemented the primary goal of using while-printing reinforcement for clay [21]. This underscores the significance of this topic within the field of architecture. The present work contributes to the existing research by offering a broader spectrum of filament materials used, additional experiments regarding mechanical properties, and the fabrication of a unique prototype.

## 2. Materials and Methods

**Figure 1:**
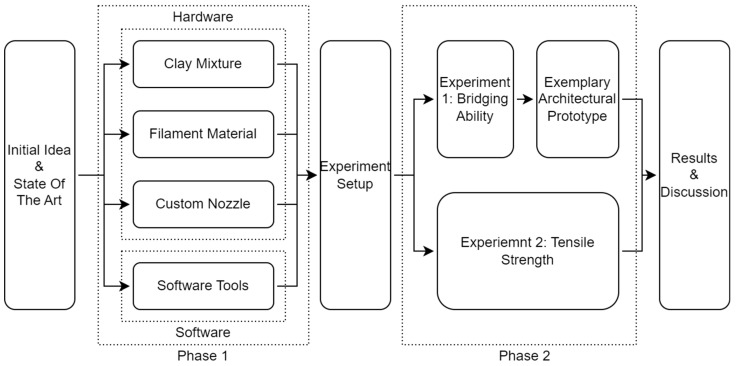
Scheme illustrating the research process.

Several experiments were carried out to validate the potential of filament-reinforced 3D printed clay and to develop its production and shaping methods. These were divided into two phases. Phase one (described in Section 2.1) consisted of (a) the preparation of a suitable clay mixture, (b) the comparison of different filament materials, and (c) the evaluation of several nozzle designs. Phase two included all results of phase one and experiments carried out to (a) evaluate the improvement of the bridging ability of wet, unfired clay (described in Section 2.3) and (b) to perform material testing of the tensile strength of dried, unfired test specimens (described in Section 2.4) and (c), ultimately, to produce an exemplary 1:1 brick module to demonstrate a potential architectural application (described in Section 2.5) (Figure 1). Methods for statistically analyzing the data (described in Section 3.2) comprise standard methods such as determination of average, standard deviation, and moving averages.

### 2.1. Hardware Setup

In order to achieve an even extrusion of clay encasing the filament material, various hardware and software parameters needed to be determined by performing a series of experiments. These parameters included a specific clay mixture, a filament material, a filament guiding nozzle, and a custom-made machine code defining the printing path. These experiments were also necessary to ensure the reproducibility and comparability of the output from both phases.

#### 2.1.1. Clay Mixture

For this study, clay powder type 209 (Goerg & Schneider, Boden, Germany) was used, consisting of 72.2% SiO_2_, 22.4% Al_2_O_3_, 2.8% K_2_O, 1.3% TiO_2_, 0.9% Fe_2_O_3_, and 0.3% MgO, as well as amounts of CaO and Na_2_O smaller than 0.1%. The clay powder, compared to clay modeling mass, allows clay mixtures to be printed while ensuring an exact moisture content in the material. Furthermore, unlike modeling mass, clay powder takes up less storage space and weighs less, making transportation more viable.

For the printing process, attempts were made to keep the moisture content of the final mixture as low as possible to decrease the chance of negative effects associated with shrinking, such as cracking and uneven alterations in geometry. The need to be able to extrude the material smoothly through the hardware components, as described in the subsequent sections, restricted the amount of water that could be used and, hence, the viscosity of the clay mixture.

The mixture was blended by using an industrial mixing machine containing a kneading arm designed for dough for a minimum of 10 min and allowed to rest for a minimum of 24 h, as an observation was made that the adhesive nature of the mixtures increased significantly after resting. The weight ratio of clay powder to water was 10:3 and was applied in phase two. Preliminary investigations of this clay material regarding viscosity revealed that the yield stress was reached at 3300 pascals. Once this point was reached, the shear stress remained constant while the shear rate increased. For the tests, a Netzsch Kinexus Prime lab+ rheometer was employed, with samples measuring 1 mm in height and tested at 20 °C.

#### 2.1.2. Filament Material

An initial experiment was carried out to cover filament materials with a clay mixture and to evaluate suitable types of filaments. A set of different filament materials with thicknesses ranging between 0.2 and 2 mm was selected based on existing studies [22,23] and preliminary work [24]. For testing purposes, each filament was positioned at the center of a circular clay extrusion with an 8mm diameter, leading to clay-to-filament cross-sectional area ratios ranging from 100:0.06 to 100:6. The filaments were tested with regard to their stiffness, elasticity, surface texture, accessibility (availability and affordability) and, above all, their ability to compound with wet clay (Table 1). The maximum diameter was limited by the nozzle design, which is explained in Section 2.1.3. After performing these initial experiments, metallic filaments (fine ropes constructed from stainless steel, obtained in 1 × 7, 1 × 19, and 6 × 7 configurations, each with an overall diameter of 0.45 mm and a tensile strength of 1770 MPa) exhibiting a repellent behavior with wet clay were excluded from further research. The high degree of stiffness and low accessibility of certain filaments also influenced the decision to discard them from this research. Viable results were obtained while using polyamide filaments with different surface textures, such as extruded polyamide, as well as with mineral filaments with rough surface textures, such as silicate filaments. Work with these filaments was continued in phase two.

#### 2.1.3. Customized Nozzle

The starting point of the hardware setup was a 3D paste-based extrusion printer for clay, a Delta WASP 40100 Clay model from the year 2020 (Massa Lombarda RA, Italy), with a maximum printable cylindrical region of 40 cm in diameter and f 100 cm in height. The 3D printer’s further specifications include a minimum layer height of 0.5 mm and a maximum printing speed of 9000 mm/m. The stepper motor-driven extruder of the printer is built for nozzle diameters between 2 and 8 mm. The nozzles, printing bed, and material tanks were custom-made.

**Figure 2:**
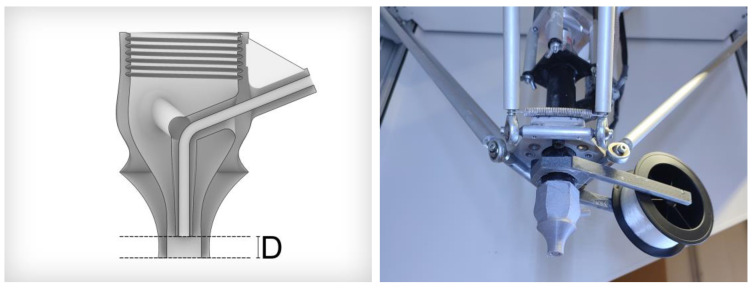
Nozzle design displayed as section drawing showing a distance (D) of 5 mm (**left**) and mounting the nozzle together with the spool holder (**right**).

The design of the filament-guiding nozzle was crucial for successfully printing the filament-reinforced clay objects (Figure 2, left). The nozzle consists of (a) a thread used to mount it tightly onto the extruder, (b) a smaller inner nozzle that guides the filament toward the center of the extrusion channel (the filament is to be fed from the outside), and (c) a larger surrounding nozzle that shapes the extruded clay. Furthermore, a spool holder was added to the printer head to ensure that the filament was unrolled easily (Figure 2, right). If unwinding the filament from the spool required too much force, the filament would shorten printing paths and thereby interfere with the geometry of the already extruded clay.

The internal nozzle configuration features a double-curved surface designed to achieve a gradual transition from the 25 mm extruder diameter to the 8 mm nozzle tip over a height of 40 mm, ensuring a smooth and continuous profile. It was aimed to minimize the internal volume of the nozzle as increased material volumes between the extruder’s auger and the nozzle’s exit would result in extrusion process delays. All interior obstacles inside the nozzle, such as the three bridges mounting the inner nozzle in the center, were placed symmetrically along a vertical center axis to ensure equal flow dynamics in all printing directions. Moreover, all interior edges were chamfered to avoid the formation of material deposits on the inside, which could dry during the printing process and clog the nozzle. 

The ratio of extruded clay to filament diameter is important, as this enables the filament to be successfully pulled along during the printing process. If the material is flowing insufficiently quickly, the filament does not have enough tension to unroll evenly. In phase two, the best results were achieved when using a nozzle with an inner nozzle diameter of 2.5 mm with a wall thickness of 0.75 mm, an outer nozzle with an 8 mm diameter, and a vertical distance D of 5 mm between the closings of the nozzles.

To eliminate the requirement for a filament feeding mechanism, it was necessary to design the nozzle in a manner that would enable the flow of clay material to naturally draw the reinforcing filament forward. After the initial tests, it became apparent that the distance (D) between the end of the inner nozzle and the end of the outer nozzle determined whether the filament would be pulled firmly enough and whether the encasing of the filament with clay would create air-filled hollow spaces or not. Efforts were invested to avoid the latter outcome, as hollow areas tended to collapse once further weight was added in the course of the printing, resulting in a loss of the desired geometry. The goal was to ensure that the clay could close its own material flow after being divided by the inner nozzle and its mounts.

**Figure 3:**
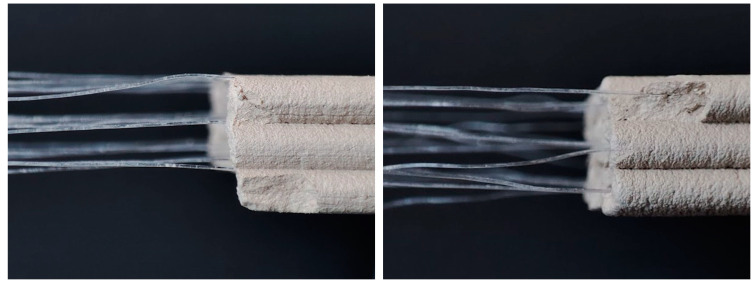
Pictures showing effects of the inner nozzle geometry on printed pieces in terms of filament placement above (**left**) and inside the extrusion (**right**). All samples were fabricated using the developed hardware setup.

The placement of the filament within the printed extrusion was also observed to be defined by the described distance (D). Having both nozzles ending at the same height causes the filament to be placed above the extrusion and results in a loose composite, which was not desired, as it would not have a positive impact on the bridging ability of single extrusions (Figure 3, left). It was aimed to position the filament at the central axis of the extrusion, which was successfully realized by having a distance (D) of 5 mm (Figure 3, right).

**Figure 4:**
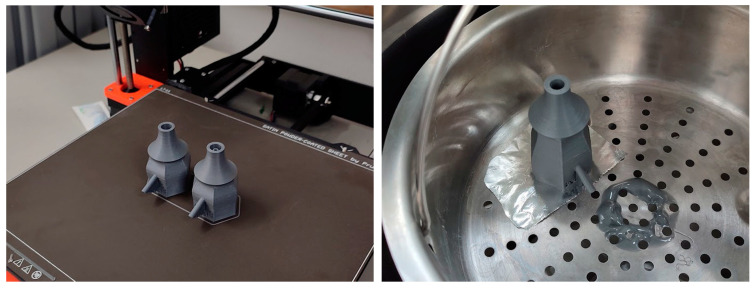
Production of ABS printing the nozzles (**left**) and smoothing them with acetone (**right**).

A Prusa i3 MK3S+ 3D printer (Prusa Research a.s., Prague, Czech Republic) with a 0.4 mm nozzle was used to produce all tested nozzles (Figure 4, left). Acrylonitrile butadiene styrene copolymers (abbreviated as ABS) with the product designation PrimaValue from 3D Prima were chosen due to their high durability, allowing for thinner nozzle parts and, thereby, larger sections of extrusion, and their ability to be smoothed. This material has a density of 1.03 g/cm^3^ and a melting point of 245 °C. 

To ensure the formation of a smooth surface on the inside, the printed nozzles were smoothed through exposure to acetone vapor [25] since any remains of surface texture originating from the layer-based additive manufacturing would have increased the friction of the clay mixture and the filament (Figure 4, right). Fibrous filaments, such as mineral fibers, tend to clog the nozzle when exposed to a rough guiding channel. This exposure was achieved by pouring 100 mL of acetone into an airtight vessel that also contained the nozzle so that the solvent covered the base of the vessel without having contact with the printed nozzle. After 15 to 30 min with no ventilation, the surface was smooth while the overall geometry had still been preserved.

### 2.2. Software Setup

**Figure 5:**
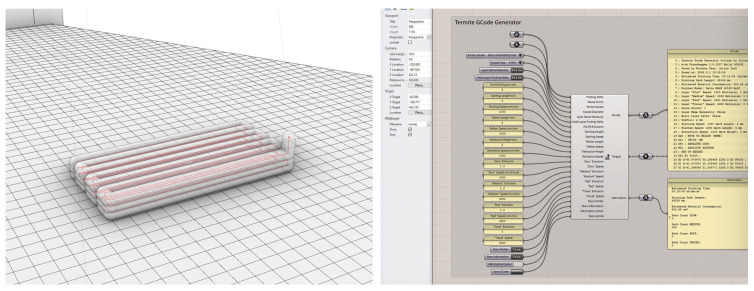
Images showing the printing paths in Rhino 3D (**left**) and the G-code generator in Grasshopper (**right**).

Rhinoceros 3D (McNeel Europe S.L. C/de Roger de Flor, 32, 08018 Barcelona, Spain) modeling software (7 SR 20) was used to provide the data regarding the movement and extrusion of the printer. Printing paths were drawn as lines either manually or parametrically by using the visual programming language Grasshopper (Figure 5, left). A digital model of the hardware setup was also used to simulate printing procedures to avoid collisions with the newly attached equipment. Based on the authors’ previous experience and knowledge about the exact material behavior, the appearance and shrinkage of the printed object could be simulated and adjusted in advance.

Developing a new software was necessary for the direct transmission of Rhinoceros 3D geometry into a G-code [26] in order to 3D print the composite material. Grasshopper was used to create a software plugin named Termite [27] that could generate G-codes containing information about the toolpath, pause positions, and the extrusion rate directly in the environment used for designing the individual objects (Figure 5, right). Thereby, designing and providing the machine data was directly achieved with a single software, allowing a highly efficient workflow. The printing paths were created through curves that were discretized and divided into parts, whereby the control points were transferred into a G-code with specific printing speeds and material flow values. A single-lined printing path was introduced for the test prints and the succeeding test specimens. At the end of each printing path, a pause function was implemented to manually cut the string and give the machine a signal to continue before returning it to its home position by inserting an unconditional stop function into the G-code. As the string had to be cut manually, all objects were designed from a continuous printing path.

### 2.3. Experiment 1: Bridging Ability

**Figure 6:**
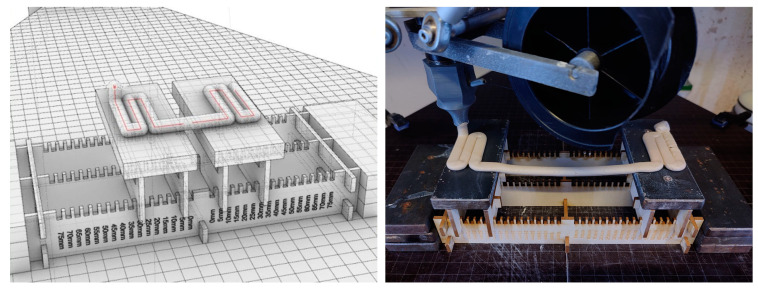
The experimental setup (**left**) increased the bridging ability during the testing procedure (**right**).

To test the bridging ability (i.e., the ability to print in mid-air), clay was printed in between two elevated printing beds separated by a configurable distance ranging from 0 to 300 mm (Figure 6, left and right). The composition of the material employed in this study remained consistent with previous experiments, featuring a clay powder-to-water weight ratio of 10:3. For reinforcing, a black corded polyamide filament was used to achieve a visual differentiation between the clay and the filament material.

To start the printing process, parameters such as the material pressure, material amount being extruded, and printing speed were set to allow for the best possible printing in mid-air. The air pressure on the material in the tank was set at a constant value of 5 bar, which enabled enough material to be fed into the extruder to achieve an appropriate amount of extrusion. The material extrusion was set to a factor supplying enough material for mid-air extrusion to produce a circular strand following the printing path with minimal sagging and to maintain an extrusion diameter of 8 mm (based on a nozzle diameter of 8 mm). Less material being extruded would have caused the extrusion to break in mid-air, while increasing the material extrusion above the ideal value would have caused the material to sag further, resulting in a mismatch between the printing path and the extruded material. The printing speed was set to 4500 mm per minute to achieve optimal results, considering the viscosity of the given material mixture. Any speed lower than this would have increased the chance of breaking the extrusion while it is being printed in mid-air, while any speed higher than this would have prevented the clay from establishing a sufficient connection to the printing bed below. After the optimal settings were determined, all tests were carried out without changing the values.

### 2.4. Experiment 2: Tensile Strength

After assessing the improvement in performance by reinforcing the wet clay during printing, the effect of the reinforcement on the mechanical properties of the material after the clay had dried was characterized. Four types of samples were tested: (1) unreinforced clay, (2) reinforced clay containing extruded polyamide filaments with a plain texture, a tensile strength of 28 N, and a diameter of 0.23 mm, (3) reinforced clay containing corded polyamide filaments with a fibrous surface, a tensile strength of 250 N, and a diameter of 0.8 mm, and (4) reinforced clay containing mineral silicate filaments with a fibrous surface, a tensile strength of 30 N, and a diameter of 0.46 mm. The latter was chosen due to its resistance up to 1400 °C.

**Figure 7:**
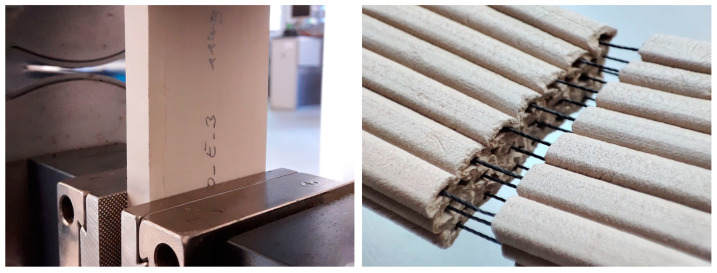
Pictures showing the testing procedure (**left**) and a sample after failure (**right**).

All testing samples had identical dimensions of 100 × 50 × 12 mm once they had been dried (approximately 48 h after printing) and sanded, including three layers of extrusion, each containing seven parallel filaments along the longer dimension of the sample. The settings for printing, such as speed, material pressure, and flow factor, had consistent values throughout the process of printing all samples. Uniaxial tensile strength tests were conducted using a Shimadzu AG-X plus testing machine ((Shimadzu, Kyōto, Japan)). The samples were mounted between two rough grippers (Figure 7, left) so that the test length, $l_{0}$, was 80 mm, and the cross-sectional area $A_{0}$ was 50 × 12 = 600 mm^2^. Loads were applied in the displacement-controlled mode, with strain rates of 25% per minute. Roughly 20 samples were tested from each type, which allowed for the statistical treatment of the results. The failure of a sample was defined as the presence of cracks in the clay, resulting in a relaxation of the composite (Figure 7, right).

### 2.5. Exemplary 1:1 Architectural Prototype

**Figure 8:**
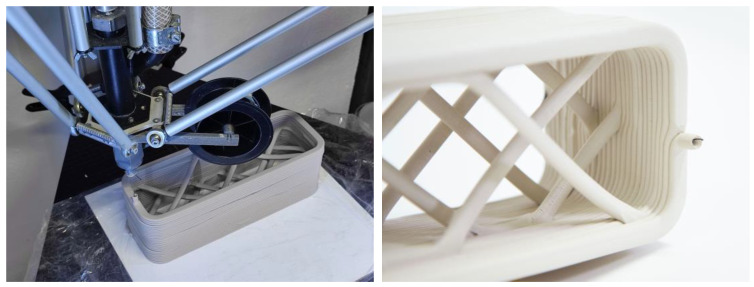
Pictures of exemplary 3D printing of a brick stiffened by reinforced bridges (**left**) and the final prototype showcasing the potential of filament-reinforced 3D printing (**right**).

Finally, to demonstrate the architectural application, an exemplary 1:1 brick module was fabricated, which would not have otherwise been possible using conventional 3D printing methods with clay. The dimensions of each module were 25.5 cm × 9 cm × 8 cm, containing 10 bridges of 11 cm and 4 bridges that were 6 cm in length, which hung freely during the printing process (Figure 8, left). Polyamide filament was used as a reinforcement. Based on the values that enabled an optimal printing result for bridging elements to be achieved, as described in Section 2.3, the speed of printing the segments in mid-air was set at 4500 mm per minute, while the speed of printing the standard layer-on-layer segments of the paths was set at 3000 mm per minute. This ensured the establishment of a safe connection between the wall and bridge elements, as well as increased the overall precision of the process. Once printed, the modules were slowly dried in a chamber to evenly dry the wall and bridge segments and to avoid cracking. This prototype represents an example of a 3D-printed brick-like module that contains an inner bracing without closed chambers as, for example, a 2D-extruded brick would necessarily have. The novel developed method clearly offers new, advantageous ways of 3D printing clay (Figure 8, right).

## 3. Results and Discussion

### 3.1. Results of Experiment 1: Bridging Ability

The following results depict findings from tests regarding the enhanced bridging capability during extrusion. As the distance between the two printing beds was increased by 5 mm for each iteration of the experiment, the maximum distance that could be bridged by this material mixture without any additional reinforcement was observed to be 50 mm. A test distance was labeled as successful once it was possible to bridge two times out of three without a significant number of cracks forming on the surface in order to ensure reliable production results. The maximum distance that could be successfully bridged by the filament-reinforced clay was 280 mm. The results show an increased bridging ability of 460% under consistent testing conditions.

**Figure 9:**
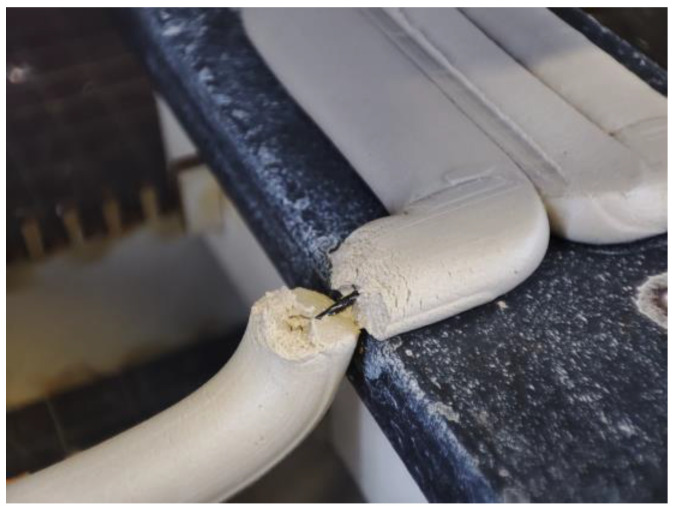
Close-up picture displaying the failure behavior during the printing process.

Cracking was observed to occur on the first printing bed’s edge at all times, as the transition from being pressed against a solid surface to being ejected in mid-air resulted in a sudden change in the pressure within the extruded material (Figure 9). This pressure change led to a large concentration of stress, which tended to break off the extrusion. If sudden cracking of the clay extrusion occurred, the filament kept the printed object in place and only allowed for minimal displacement. This finding suggests that otherwise brittle, clay-based building elements could be given the quality of non-catastrophic behavior by reinforcing them with filaments, protecting them in the event of a collapse. Furthermore, having continuous reinforcement within the extrusion was shown to decrease the chance of crack formations during drying, while the overall stability of the printed structure was increased.

### 3.2. Results of Experiment 2: Tensile Strength

**Figure 10:**
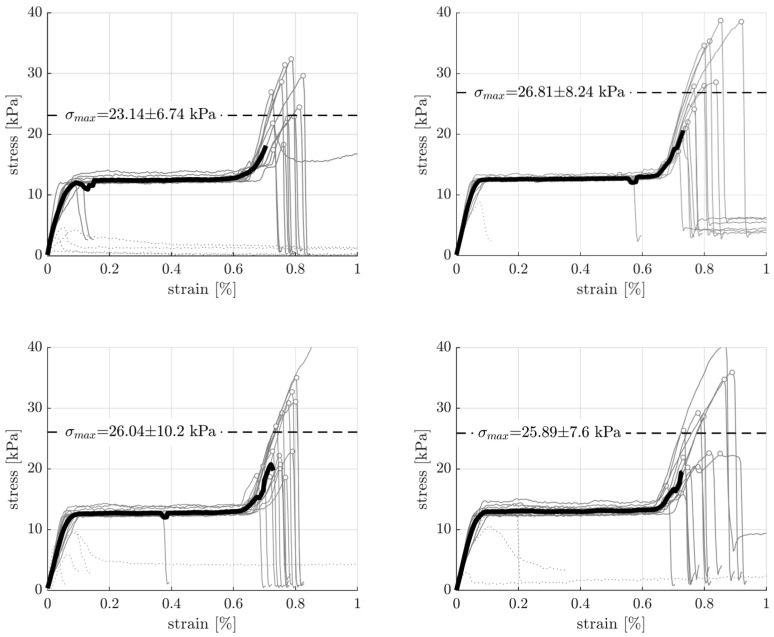
Stress-strain diagrams for the unreinforced (**top left**) as well as reinforced samples with plain polyamide filaments (**top right**), corded polyamide filaments (**bottom left**), and mineral silicate filaments (**bottom right**), showing the individual curves (gray lines) and a moving average (black bold line). Reinforcing increases the tensile strength by roughly 15% and additionally reduces the number of outlier samples (dotted gray lines) with rather low strengths due to pre-existing cracks.

Following the drying of test specimens, an evaluation was conducted to assess the impact of reinforcement on the mechanical properties of the material. The uniaxial tensile stresses are obtained by dividing the recorded tensile force, F, by the cross-sectional area $A_{0}$, $\sigma =F/A_{0}$. Uniaxial tensile strains, ε, arise from the applied displacements, Δl, as $\varepsilon ={\Delta l/l}_{0}$. In this way, stress-strain diagrams (σ-ε diagrams) were obtained and analyzed (Figure 10). Interestingly, after observing an initial, virtually linear increase in the stress as the strain increased, a plateau was detected at stress levels of around 13 kPa for both the unreinforced and the reinforced samples. After the plateau had been reached, the stresses increased again until failure occurred. The mechanical reason for the observed plateau is not clear. Compression or shear tests performed on raw clay reveal a similar plateau [28,29], but the increase seen afterward in this work has not been detected in other studies. Structural effects may play a role here, such as the clamping of the samples, as the fracture mostly occurred close to the clamps or re-alignment of the filaments at the clamped ends. Moreover, a slip between the machine and the sample may have occurred.

After discussing the qualitative shape of the σ-ε diagrams, a quantitative comparison between the unreinforced and reinforced samples was performed. The average tensile strength (=maximum stress), $\sigma_{max}$, of the unreinforced samples was 23.14 kPa, with a standard deviation of 6.74 kPa. The reinforced samples appear to be slightly stronger, whereby the type of reinforcement had only a very minor effect on the tensile strength. Described in more detail, the strength of the samples with straight polyamide filaments is the highest (26.81 ± 8.24 kPa), followed by the samples with corded polyamide filaments (26.04 ± 10.2 kPa), followed by those with the mineral silicate filaments (25.89 ± 7.60 kPa). These results show that the average tensile strength of the reinforced sample could be improved by roughly 15%.

Notably, outliers were removed from the calculations of averages, standard derivations, and moving averages. A stress-strain curve is considered an outlier if the sample fails before reaching the plateau. In mathematical terms, all tests for which the maximum stress measured is below the plateau stress of 13 kPa are considered outliers. Regarding these samples, failure occurred at much lower maximum stresses and, more noticeably, at much lower ultimate strain levels, and sometimes even before any load had been applied. Pre-existing microcracks resulting from the severe shrinkage might be one reason for the observed early failure. Interestingly, as many as five outliers were observed for the unreinforced samples, while only one was observed for the samples reinforced with straight polyamide filaments. These results show that the reinforcement also helps to prevent these cracks and supports the 3D-printed structure during the drying process.

**Figure 11:**
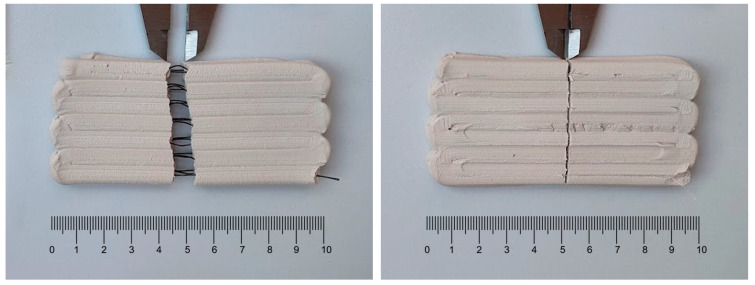
Comparison between crack openings after failure for corded polyamide-reinforced composite with a rough surface (**left**) and plain polyamide-reinforced composite with a flat surface (**right**).

Finally, an interesting observation can be reported when comparing the behavior of the samples with corded polyamide filaments with the behavior of those with plain polyamide filaments. After failure (which occurred at roughly the same stress levels in both), the crack opening for samples with the corded filaments (Figure 11, left) is more than one order of magnitude larger than the crack opening observed for samples with the plain filament (Figure 11, right). This results from the debonding of the corded filament from the clay, which creates an essentially hollow, cylinder-like structure.

## 4. Conclusions

In order to integrate reinforcement into the 3D printing of clay, we successfully developed hardware and software components that can be used to produce 3D-printed clay objects, including a while-printing linear filament reinforcement. The study findings support the conclusion that filament-reinforced 3D printing of clay is a viable solution that can be applied to increase the tensile strength of unfired clay in both a wet and a dry state. The filament material can be chosen based on the object’s final purpose, ranging from increased support while drying to allow for the production of more lightweight constructions to increased tensile strength in the dried clay objects. The best results regarding multiple criteria were achieved when using extruded polyamide filament-reinforced clay with a plain texture and a diameter of 0.23 mm.

The new method increased the ability of linear extrusions to span bridges while printing by 460%. Furthermore, when comparing the unfired clay test specimens without and with while-printing filament reinforcement, an increase of roughly 15% in the tensile strength values along the axis of extrusion was observed. 

Currently, the main limitation of the newly developed method is the necessity to have one continuous printing path. Further, once the lifespan of the composite has ended, the material needs to be separated into its individual components, making up- or recycling purposes more difficult. However, linear while-printing reinforcement presents an alternative to the conventional methods used to increase tensile strength, such as adding fibers to the clay mixture. Industrial production seems feasible for more versatile lightweight designs, including bracing structures, in a high-performance sector of the building industry.

## 5. Outlook

If this research is contextualized regarding its potential for future implementation, the developed building principle clearly has the potential to be used to construct large-scale, freeform structures. The enhancement in bridging ability enables the 3D printing of more structurally efficient objects with added bracings, as well as facilitating the creation of more porous designs. Examples of this capability can be observed in Section 2.5 and in the woven clay extrusions developed at Harvard University [30]. In this study, additional research directions and questions were identified.

Further research has to be conducted to determine how polyamide filaments, such as extruded polyamide with a plain texture, are able to handle clay shrinkage without exhibiting large displacements after breaking. The overall aim of such research would be to adapt the gained knowledge to other more sustainable filament materials, such as natural fibers [31,32,33], in order to address known recycling issues of composite materials [34,35]. As filaments with a plain texture have proven to be more suitable, further material testing is planned, including tests with optical fibers such as glass fibers, polycarbonate, or polymethyl methacrylate.

The next step will be to introduce an automated filament-cutting mechanism, which will be controlled by embedding additional signals in the G-code. Once the process is fully automated, the necessity to compose the design in one continuous printing path will be eliminated. Furthermore, it is conceivable to incorporate a sewing-like mechanism to enhance the tensile strength between layers [36].

To increase the tensile strength of fired high-performance elements, a high temperature-resistant filament, such as materials used for the aerospace industry with a tensile strength exceeding the one of fired clay, needs to be found with reasonable accessibility. 

**Figure 12:**
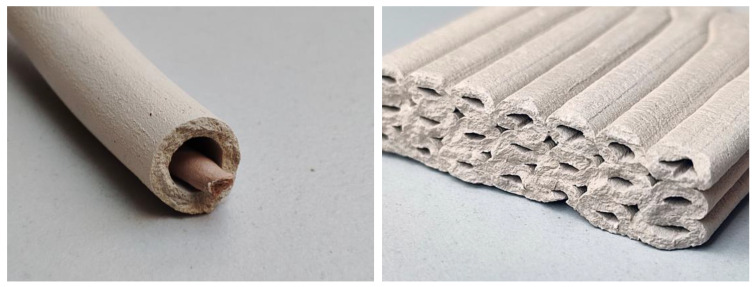
Possible alternatives to using the filament-guiding nozzles to insert other materials, such as flammable organic matter (**left**) or to print hollows (**right**).

Another notable finding that emerged from this research is the use of the method developed for filament-reinforced printing to introduce other materials, such as lightweight porous foam, flammable organic matter, or gel-like support material [37], through the inner nozzle in order to fill the center volume of the extrusion (Figure 12, left) resulting in a co-extrusion process [38]. It is conceivable that such a process enables controlled material distribution within the extrusion [39]. Preliminary tests involving different clay types, flammable filaments to produce continuous cavities (Figure 12, right), and organic substrates for mycelial growth [40] have been carried out and successfully tested for their applicability.

## Figures and Tables

**Table 1:** Comparison of all filament materials according to the following criteria: adhesion, stiffness, elasticity, texture, and accessibility.

Thread Type	Adhesion	Stiffness	Elasticity	Texture	Accessibility
Synthetic	Low	Low	Low	Medium	High
Cotton	High	Low	Medium	Medium	High
Nylon	Low	Medium	Medium	Plain	High
Carbon	Medium	Low	Low	Rough	Medium
Glass	Medium	Low	Low	Rough	Medium
E-Glass	Medium	Low	Low	Rough	Medium
Basalt	Medium	Low	Low	Rough	Medium
Stainless Steel	Low	High	Low	Medium	Low
Silicate	Medium	Low	Low	Rough	medium

## Data Availability

Data available on request.
